# The Estimates of the Health and Economic Burden of Dengue in Vietnam

**DOI:** 10.1016/j.pt.2018.07.007

**Published:** 2018-10

**Authors:** Trinh Manh Hung, Hannah E. Clapham, Alison A. Bettis, Hoang Quoc Cuong, Guy E. Thwaites, Bridget A. Wills, Maciej F. Boni, Hugo C. Turner

**Affiliations:** 1 Oxford University Clinical Research Unit, Wellcome Trust Major Overseas Programme, Ho Chi Minh City, Vietnam; 2 Centre for Tropical Medicine and Global Health, Nuffield Department of Medicine, University of Oxford, Oxford, UK; 3 London Centre for Neglected Tropical Disease Research, London, UK; 4 Department of Infectious Disease Epidemiology, School of Public Health, Faculty of Medicine, St Marys Campus, Imperial College London, Norfolk Place, London W2 1 PG, UK; 5 Pasteur Institute, Ho Chi Minh City, Vietnam; 6 Center for Infectious Disease Dynamics, Department of Biology, Pennsylvania State University, University Park, PA, USA

**Keywords:** dengue, health burden, economic burden, cost of illness, Vietnam, DALYs

## Abstract

Dengue has been estimated to cause a substantial health and economic burden in Vietnam. The most recent studies have estimated that it is responsible for 39 884 disability-adjusted life years (DALYs) annually, representing an economic burden of US$94.87 million per year (in 2016 prices). However, there are alternative burden estimates that are notably lower. This variation is predominantly due to differences in how the number of symptomatic dengue cases is estimated. Understanding the methodology of these burden calculations is vital when interpreting health economic analyses of dengue. This review aims to provide an overview of the health and economic burden estimates of dengue in Vietnam. We also highlight important research gaps for future studies.

## Highlights

The estimates of the total number of symptomatic dengue cases occurring annually in Vietnam have increased significantly over time, largely due to changes in the methodology used to adjust for underreporting.

The estimates of the total health and economic burden of dengue vary significantly, mainly due to differences in the estimated annual incidence of symptomatic dengue cases.

The DALY calculation for dengue has changed significantly over the last decade.

Without understanding the methodology used to estimate the health and economic burden of dengue, it is not possible to critically examine health economic analysis of dengue interventions.

## Dengue Burden

Dengue is a mosquito-borne disease occurring in over 100 countries in Asia, the Pacific, the Americas, Africa, and the Caribbean [1]. Symptomatic infection most commonly presents as a mild to moderate, acute febrile illness. However, as many as 5% of dengue cases develop severe life-threatening disease known as severe dengue 2, 3.

The global incidence of dengue has increased notably over the last 50 years, and in 2015 over 3.2 million cases from the Americas, South-East Asia, and Western Pacific regions were reported to the World Health Organization (WHO)i [4]. However, many cases are not reported to national health systems, and additional methodologies are needed to estimate the true burden of dengue. The estimates of the average true number of **symptomatic dengue cases** (see Glossary) occurring globally vary between 58 and 101 million per year 5, 6, 7.

Accurate knowledge of a disease’s health and economic burden is important for supporting the development of efficient public health policies, and for helping to guide the allocation of limited healthcare resources. Within the Global Burden of Disease (GBD) 2013 study, it was estimated that dengue was responsible for 1.14 million **disability-adjusted life years (DALYs**) globally, with a corresponding global economic burden of US$8.9 billion in 2013 prices 6, 7. However, estimates of dengue’s health and economic burden vary between different studies and the approaches used have changed over time 6, 7, 8, 9. This review aims to provide a critical overview of the current estimates within the literature relating to dengue’s health and economic burden in Vietnam. Specifically, we summarise:

(i) the number of dengue cases reported to the health system and the different estimates for the true number of symptomatic cases occurring;
(ii) the estimates of dengue’s DALY burden, and how the DALY calculation for dengue has changed over time;
(iii) the reported costs relating to dengue cases (adjusted for inflation), and the current estimates of its total annual economic burden;
(iv) key areas for future research.

Although this paper focuses on dengue in Vietnam, many of the issues are relevant for all dengue health and economic burden calculations, as well as more generally for calculations for other diseases.

## The Number of Symptomatic Dengue Cases in Vietnam

### The Number of Reported Cases

**Figure 1:**
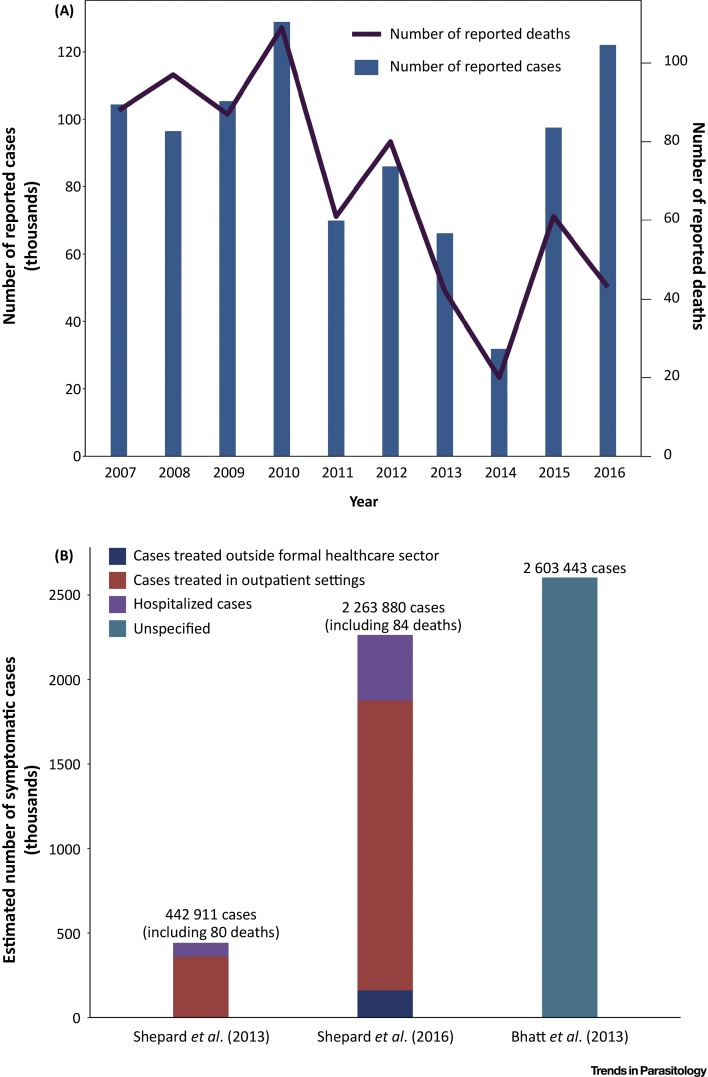
The Number of Reported Dengue Cases and the Estimates of the True Number of Symptomatic Dengue Cases Occurring per Year in Vietnam. Panel A illustrates the number of reported dengue cases in Vietnam over time (the data are reproduced from 88, 89, 90, 91, 92, 93, 94). However, many symptomatic dengue cases are not reported and therefore these values underestimate the true burden of dengue. Panel B illustrates the different estimates regarding the true number of symptomatic dengue cases occurring annually in Vietnam (Shepard *et al*. (2013) [9], Shepard *et al*. (2016) [6], Bhatt *et al*. (2013) [17]). The Shepard *et al*. [9] estimates are representative of the period between 2001 and 2010. The Shepard *et al*. [6] estimates are based on the GBD 2013 study (although the model used smoothed out the effects of dengue outbreaks, making the estimates more representative of an average year around 2013). The Bhatt *et al*. [17] estimate pertains to 2010. The different case categories are defined in the Glossary. A more detailed description of the data is given in Table S1.

In Vietnam, the number of reported dengue cases varies significantly year by year (Figure 1A). Between 2007 and 2016, the average number of reported cases per year was 90 844. Dengue outbreaks tend to be larger and more frequent in the southern provinces, with the incidence of infection typically peaking between June and Octoberii.

In Vietnam, dengue is a notifiable disease and clinically suspected and confirmed dengue fever cases have to be reported to the Ministry of Health 10, 11. However, although the reporting system operates throughout the country, in practice most nonhospitalized (outpatient) cases, as well as many of the cases that are treated within the private sector, are not reported to the Ministry of Health [12]. It is also possible that some hospitalized dengue cases are misdiagnosed. Consequently, the number of reported cases does not reflect the true number of symptomatic dengue cases occurring. As improvements are made to the reporting systems, it is likely that more of the nonhospitalized cases will be reported.

Currently, there is no system to check or validate the cause of death in Vietnam. It is therefore possible that the number of dengue-related deaths is also underreported. This is supported by recent studies which have shown that dengue-related deaths can still be underreported in countries with better-funded surveillance systems 13, 14.

### The Estimated Total Number of Symptomatic Cases

The different estimates of the true number of symptomatic dengue cases occurring annually in Vietnam (after adjusting for underreporting) are outlined in Figure 1B and in Table S1 in the supplemental information online. In 2013, the Shepard *et al.* [9] estimate of 442 911 symptomatic cases per year was derived by multiplying the number of officially reported cases with a Vietnam-specific **expansion factor** of 5.8 15, 16 (Box S1 in the supplemental information online). This annual estimate was representative of the period between 2001 and 2010. In contrast, in 2016 a new estimate of 2 263 880 symptomatic cases occurring in 2013 was reported [6]. This was based on the methodology used within the GBD 2013 study [7], where the underreporting was adjusted for using a modeling approach described in Stanaway *et al*. [7]. This updated estimate was based on data from a wider reference period than previously used (1988–2013 vs. 2001–2010) and included an estimate for the number of patients treated outside of the formal healthcare sector [6]. The model used smoothed out the effects of dengue outbreaks, making the estimate more representative of an average year around 2013. It should be noted that, although this new estimate was based on the approach used within the GBD 2013 study [7], there were some differences in the methods and the reported estimates – for example, the estimated number of dengue-related deaths in Vietnam within the GBD 2013 study [7] was higher (278 vs. 78).

In contrast, using a geospatial modeling framework to map the global distribution of dengue risk, Bhatt *et al*. [17] estimated that, in 2010, there were 7 965 912 asymptomatic and 2 603 443 symptomatic dengue cases in Vietnam (Figure 1B and Table S1). Although the Bhatt *et al*. [17] estimate of the total global number of symptomatic cases was higher than that from the GBD 2013 study [7] – 96 million (95% credible interval (CI): 67–136 million) vs. 58.4 million (95% uncertainty interval (UI): 23.6–121.9 million) – the estimates for Vietnam were similar (Figure 1B and Table S1).

When comparing these estimates, it is also important to consider that the number of symptomatic dengue cases occurring will likely increase over time due to population growth, and possibly due to increases in transmission rates.

It should be noted that, as research groups are aware of previous estimates, there is a risk that this could bias their study in terms of influencing their methodology and the interpretation of their results.

## The Estimated DALY Burden of Dengue in Vietnam

The health burden of dengue is often summarized in terms of DALYs. DALYs are calculated as the sum of two components: the years of healthy life lost due to disability, and years of life lost due to premature mortality 18, 19. It therefore combines mortality and morbidity into a single metric, and one DALY can be thought of as 1 year of healthy life lost.

Within the GBD 2013 study [7] it was estimated that, in 2013, dengue was responsible for 39 884 DALYs in Vietnam. The years of healthy life lost due to disability represented 55% of this estimate, with the years of life lost due to premature mortality accounting for the remaining 45%. This was approximately four times the previous estimate from Shepard *et al*. [9] of 11 079 DALYs being lost per year. A key reason for this difference is the higher updated estimations of the typical annual incidence of symptomatic dengue cases made within the GBD 2013 study (which used an updated method for estimating the incidence of symptomatic dengue cases) (Figure 1B). In addition, the general methodology used for DALY calculations (i.e., not just for dengue) has changed over time (outlined in Box 1). The different estimates of the DALY burden of dengue in Vietnam are outlined in Table S2.

Box 1 **Overview of the Key Changes Made to the DALY Framework over Time**

Up to 2013, the global health field relied heavily on the set of disability weights derived from the 1996 version of the GBD 1990 study and its subsequent 2004 revision 61, 62, 63. These disability weights were developed by a small panel of health professionals by using two different person trade-off questions (such as comparing the value of extending the life of healthy individuals to that of individuals with a particular disabling condition) 22, 64, 65. The weights were intended to reflect societal judgments regarding the value of averting different diseases and not individual judgments of the burden of the diseases themselves [66], and the weights were often specific to a given disease/sequela. The GBD 1990 DALY calculation and use of the person trade-off method were subsequently criticized 65, 67, 68, 69, 70, 71, 72. In 2007, the Bill & Melinda Gates Foundation provided funding for a new GBD 2010 study, led by the Institute for Health Metrics and Evaluation (University of Washington) [23] and their updated approach made several changes 23, 72, 73:

• The disability weights were no longer estimated using the ‘person trade-off’ method. Within the new approach 25, 61, 74, simple paired-comparison questions were used, where the respondents were asked to consider two hypothetical individuals characterized by different functional limitations, and asked to indicate which person they would regard as ‘healthier’.
• The emphasis changed from surveying health professionals to a cross-section of the general population. The GBD 2010 study performed population-based household surveys in five different countries (Bangladesh, Indonesia, Peru, Tanzania, and the USA). This was also supplemented by an open-access web-based survey [74]. In addition, the GBD 2013 collected further data from four European countries [25]. Consequently, the weights changed between GBD 2010 and 2013 studies.
• The conceptual thinking regarding how the disease burden and disability weights are defined has changed over time. Within the original GBD commissioned by the World Bank, disease burden was defined in terms of loss of well-being [75]. However, the definition gradually shifted to referring only to departures from ‘optimal health’ [23]. This change was fully implemented within the GBD 2010, where the surveys explicitly framed the questions in terms of ‘who is healthier’ 23, 25, 74. Consequently, the updated disability weights are now intended to be solely measures of losses of ‘health’ and are not intended to represent losses of well-being/welfare.
• Critics have argued that disease burden should be quantified in terms of overall welfare loss and that only measuring burden as ‘lost health’ may lead to biases when estimating the disability weights 66, 72, 76. For example, using this ‘narrow’ definition may mean that respondents undervalue the burden associated with permanent long-term disabilities (such as blindness) which are not necessarily associated with illness or ‘poor health’, despite their potential impact on the patients’ lives 23, 66, 72.
• Before the GBD 2010 study, DALY calculations typically incorporated age-weighting, which gave less weight to years of healthy life lost at young ages and older ages 22, 23, and discounted the estimated number of years of life lost (reducing their value – see Table S3). The GBD 2010 framework removed this age-weighting and discounting from their DALY calculation [24].

Due to the changes in the methodology in the different GBD studies, the estimates from these studies are often not directly comparable. To account for this, each GBD study back-calculates the burden back to the year 1990 – showing trends in burden over time with a consistent methodology. The most recent GBD results are available onlineiii.

Alt-text: Box 1

### Estimating the Years of Life Lost Due to Premature Mortality

Within the GBD 2013 study [7] the true number of dengue-related deaths was estimated using data from the GBD Cause-of-Death database and the Cause-of-Death Ensemble Model tool 20, 21. The number of years of life lost due to premature death was then calculated from the difference between the number of people dying at a certain age and their corresponding life expectancy (resulting in the number of years of life lost) [7]. The assumed life expectancy was based on a theoretical composite life table where the life expectancy for each age is equal to the longest recorded life expectancy among people of that age in any country [20]. Prior to the GBD 2010 study, the GBD used a different gender-specific standard reference life table (Table S3) [22]. A limitation of using the Cause-of-Death Ensemble Model tool is that there are notable data gaps for several large, high-incidence countries, and it could therefore be underestimating the true number of dengue-related deaths [7].

Within the Shepard *et al*. study [9], a different approach was used and the assumed life expectancy was estimated from WHO life tables for the examined endemic countries, which would generally be lower than the GBD’s theoretical composite life table. In addition, the estimated number of years of life lost were discounted into the future. This process significantly reduces the estimated number of years of life lost within a DALY calculation [23]. For example, within the GBD 1990, the projected number of years lost when a new-born male child died was 33.27 when applying age-weighting and discounting. However, this would increase to 79.94 if the age-weighting and discounting were removed (Table S3). Although discounting was recommended at the time of the Shepard *et al*. [9] study, it has been removed from the standard DALY calculation since the GBD 2010 study, increasing the estimated number of years of life lost due to premature mortality from dengue [24] (Box 1).

### Estimating the Years of Healthy Life Lost Due to Disability

Within a DALY calculation, the years of healthy life lost due to disability are calculated using a **disability weight** factor ranging between 0 and 1, which reflects the severity of the disease and sequelae – with 0 representing perfect health and 1 representing death. The different disability weights that have been used for dengue DALY calculations have changed significantly over time (outlined in Box 2). Interestingly, many economic evaluations have used a disability weight that is not officially designated for dengue within the updated GBD studies (Box 2).

Box 2 **Summary of the Disability Weights Used for Dengue**

Below we provide a summary of the disability weights (DWs) used for dengue over time, where 0.0 corresponds to good health with no loss of well-being, and 1.0 corresponds to death:

**GBD 1990 (1996 Revision)**

Focused only on dengue hemorrhagic fever:

• The average duration was assumed to be 30 days, with an average DW of 0.2 54, 77.

**GBD 2004 Update**

Included both dengue fever and dengue hemorrhagic fever/dengue-shock syndrome:

• 94% of the symptomatic cases were assumed to have dengue fever with a mean duration of 5.5 days, and were assigned an average DW of 0.21 54, 62.
• 6% of the symptomatic cases were assumed to have dengue hemorrhagic fever/dengue-shock syndrome with an average duration of 11 days, and were assigned a DW of 0.5 (the same overall disability score as assumed within the GBD 1990, that is, using a higher DW but for a shorter duration) 54, 62.

**GBD 2013**

Assigned symptomatic dengue cases into two acute health states:

• 94.5% were assigned the DW for a moderate acute infectious disease episode (DW = 0.051) with a mean duration of 6 days 7, 25.
• 5.5% were assigned the DW for a severe acute infectious disease episode (DW = 0.133) with a mean duration of 14 days 7, 25.

In addition, 8.5% of the dengue symptomatic cases were assumed to have post-dengue chronic fatigue and assigned the DW for ‘infectious disease-post-acute consequences’ (DW = 0.219), with a mean duration of 6 months.

**Other Studies**

In a study in 1998 (before the GBD 2004 update), Meltzer *et al*. [78] used a higher DW of 0.81 for symptomatic dengue cases but assumed shorter durations of illness (based on clinical data):

• Dengue fever, dengue with severe manifestations (but not requiring hospitalization) and hospitalized cases were assumed to have an average duration of 4 days, 10 days, and 14 days respectively [78].
• The DW was based on the disability scores within the class five severity level defined within the original GBD study commissioned by the World Bank (‘needs assistance with instrumental activities of daily living such as meal presentation, shopping or housework’) 75, 78.

Subsequent studies have also used this higher DW 9, 79, 80, 81, 82, 83, 84 – though the assumed duration of illness varied.

Alt-text: Box 2

Since the GBD 2010 study, the disability weights used are no longer specific to dengue but are general weights for an acute episode of an infectious disease – stratified by severity (Box 2). Specifically, since the GBD 2013 study the healthy life years lost due to disability have been estimated as follows 7, 25:

• 94.5% of symptomatic dengue cases were assigned a disability weight of 0.051 with a mean duration of six days;
• 5.5% of symptomatic dengue cases were assigned the disability weight of 0.133 with a mean duration of 14 days;
• in addition, 8.5% of symptomatic dengue cases were assumed to have post-dengue chronic fatigue and were assigned a disability weight of 0.219, with a mean duration of 6 months.

The proportions of the different case types were applied universally to every country. However, in reality these could vary depending on each country’s local epidemiology and transmission history.

Adding this assumed level of post-acute consequences has significantly increased the estimated years of healthy life lost due to disability resulting from dengue within the GBD studies 7, 25. This was based on a study by Teixeira *et al*. [26]. However, other studies have not found the same degree of post-acute consequences, and this requires further investigation [27].

In contrast, the previous estimate from Shepard *et al*. [9] used a disability weight of 0.81 (which has been used in a range of studies – Box 2) for both hospitalized and ambulatory cases, with an average duration of 14 days for hospitalized patients, and 4.5 days for ambulatory patients.

## The Estimates of the Economic Burden of Dengue Illness in Vietnam

The fundamental goal of costs of illness calculations is to evaluate the economic burden that the illness imposes on society – including **direct medical costs, direct nonmedical costs**, and **indirect costs**. This is typically calculated by multiplying the estimated number of the different types of symptomatic dengue cases by the corresponding cost of each type of case. In the following section, we outline these reported case costs (or ‘unit costs’ in economic terms) related to dengue in Vietnam.

To make the reported costs collected in different years directly comparable, it was necessary to adjust for inflation. This was done using inflation rates relevant to Vietnam, as outlined in the supplemental information. Note that other studies have used different methods, such as using US inflation rates, and therefore some of our reported values differ from those presented in other reviews despite being based on the same original data.

It should be highlighted that there are limitations associated with adjusting costs for inflation, which are discussed further in the supplemental information.

### The Reported Unit Costs Related to Hospitalized Cases

We identified seven studies that reported costs related to hospitalized dengue cases in Vietnam. The reported average cost per case varied between US$115 and US$278 in 2016 prices (Table 1) 28, 29, 30, 31, 32, 33, 34. The average total cost per case was relatively consistent across the different studies, with the exception of the estimate from Tam *et al*. [34] which was significantly higher. A potential reason for this discrepancy could be recall bias, as questionnaires used within this study [34] were administered 6–9 months after the patients’ recuperation.

**Table 1:** Reported Costs Related to Hospitalized and Outpatient Dengue Cases (2016 US$ Prices)

Study	The sample	Direct medical costs per case (US$)^a^, ^b^	Direct non-medical costs per case (US$)^a^, ^b^	Indirect costs per case (US$)^a^, ^b^	Total cost per case (US$)^b^
Hospitalized cases					
Harving and Rönsholt [33]	The families of children hospitalized with dengue hemorrhagic fever at a hospital in Ho Chi Minh City were interviewed regarding their out-of-pocket expenses.	61.40 (excluding the costs covered by insurance schemes)	29.73	23.97	115.10
Tam *et al.*[34]	Dengue hemorrhagic fever patients admitted to two hospitals in Can Tho province were interviewed 6–9 months after their recovery.	128.00	86.06	64.00	278.06
Luong *et al.*[29]	A multicenter cost study in four hospitals in southern Vietnam. Both urban and rural settings were sampled.	71.82	26.17	19.62	117.61
Pham *et al*. [28]	Patients from one hospital in a suburban area of Ho Chi Minh City were interviewed. Direct medical costs were also collected from the hospital’s electronic database.	46.88	40.91	50.76	138.55
Stahl *et al*. [30]	A sample of patient records collected from one hospital.	45.32 (excluding staff costs)^c^	NA^d^	NA	NA
Vo *et al*. [31]	Direct medical costs were collected from the electronic database from a hospital in Ho Chi Minh City.	50.12	NA	NA	NA
Lee *et al*. [32]	Hospitalized patients from the Khanh Hoa General Hospital (Khanh Hoa province).	77.60	47.68	62.64	187.92
Outpatient (ambulatory) cases					
Luong *et al*. [29]	A multicenter cost study in four hospitals in southern Vietnam. Both urban and rural settings were sampled.	15.08	18.18	15.16	48.42
Stahl *et al*. [30]	A sample of patient records collected from one hospital.	28.46	NA	NA	NA
Lee *et al*. [32]	Suspected dengue cases visiting the outpatient clinic at Khanh Hoa General Hospital (Khanh Hoa province).	24.30	8.41	25.24	57.95

a The cost categories are defined in the Glossary.

b All of the presented results have been adjusted to 2016 prices. Any adjustments made to the original data are outlined in the supplemental information. The corresponding results expressed in international dollars are shown in Table S5.

c Also reported the following costs in 2016 prices: US$55.86 per adult general ward inpatient; US$48.49 per child general ward inpatient; US$113.84 per intensive care unit patient.

d NA, not available.

The cost of a hospitalized case increased significantly with the severity of the patient’s illness [29] (Table 1). For example, the costs for a hospitalized dengue-shock-syndrome patient were estimated to be two to three times those of a hospitalized dengue fever case 29, 33. This highlights how the relative number of severe cases within a sample may influence the reported average cost per hospitalized case. The relationship between the age of the patient and the cost of case management was inconsistent across the different studies, and overall there was no significant difference in the estimated unit costs of pediatric vs. adult cases.

It should be noted that the different costing studies captured varying inputs and collected data over varying time-frames, hindering comparisons. For example, not all of the studies collected the costs related to care that the patient may have received before they were hospitalized. Pham *et al*. [28] found that over 80% of their sample sought care at private clinics or pharmacies before they were hospitalized, incurring an average cost of US$32.05 in 2016 prices. In addition, the authors observed that many of the patients continued to incur nonmedical and indirect costs after they were discharged from the hospital [28]. This indicates that the time-frame over which the costs are collected could account for a notable degree of the variation in the costs of dengue illness reported by different studies.

The direct medical costs consistently made the largest contribution to the total cost per hospitalized case (between 46% and 61%) (Table 1). Although, the patient’s nonmedical costs remained significant. Since 2009, the Vietnamese government’s (public) health insurance program has covered at least 80% of the direct medical costs of insured hospitalized dengue patients [35]. However, the program does not compensate the patients for their direct nonmedical costs (such as transportation) or their indirect costs (lost income/productivity). Thus, there is still a significant economic burden even for individuals/families with health insurance, and as indicated in Table 1, hospitalized patients do incur notable indirect costs. There was variation across the different studies regarding how the productivity losses were quantified.

### The Reported Unit Costs Related to Outpatient Cases

The reported average cost related to cases seeking care at outpatient clinics within governmental hospitals varied between US$48 and US$58 in 2016 prices (Table 1). For this type of outpatient care, the patient's direct nonmedical costs and their indirect costs were the major contributors to the overall cost per case (Table 1). Importantly, these values represent the overall economic burden of outpatient cases and not only the costs associated with seeking treatment at the clinic (for example, they include the indirect costs associated with the patient being unable to attend work for the duration of their illness).

The studies costing outpatient cases sampled patients visiting clinics within governmental hospitals. However, many outpatients with suspected dengue prefer to visit their local doctor, which is a potentially cheaper option than an outpatient clinic at a hospital. Consequently, the costs associated with outpatient cases in cost of illness studies may be being overgeneralized, potentially overestimating the total costs related to outpatient cases.

### The Reported Unit Costs Related to Cases Treated Outside the Formal Healthcare Sector

We found very little published data regarding the costs in Vietnam of informal medical care for dengue, such as the costs associated with patients treating themselves at home with medications obtained from pharmacies or traditional medicine practitioners. Recently, Shepard *et al*. [6] projected that the average cost of informal medical care for dengue in Vietnam was approximately US$15.16 per case in 2016 prices (Table S4). However, this information is based on cost data extrapolated from other countries.

### The Reported Estimates of the Total Annual Cost of Dengue Illness

Based on the dengue incidence estimates from the GBD 2013 study, a recent analysis estimated that the total annual cost of dengue illness in Vietnam is US$94.87 million in 2016 prices. The estimates pertaining to Vietnam are summarized in Table S4 [6].

Although the assumed cost per hospitalized case was higher than that of a case treated in an outpatient facility (US$80.88 vs. US$33.36 in 2016 prices), the total cost related to outpatient care was ultimately larger (US$31.51 million vs. US$57.44 million in 2016 prices) [6]. This is because there are significantly more outpatients than inpatients (Figure 1B and Table S1) [6]. The costs can be further stratified by direct and indirect costs (US$66.48 million vs. US$28.39 million, respectively, in 2016 prices) [6]. This breakdown is important as the costs related to outpatient cases and the indirect costs were notable drivers in the total cost of illness. However, the data surrounding these costs are subject to a number of limitations (discussed further in the data gaps and future research needs section). The unit costs of hospitalized and outpatient cases we identified (Table 1) were higher than assumed in the analysis of Shepard *et al*. [6] (Table S4). This was partly due to differences in the methods used to adjust for inflation.

Within the Shepard *et al*. [6] study, the estimated costs associated with fatal cases were significantly higher than those associated with nonfatal cases (Table S4). The economic burden of fatal cases was estimated using the **human capital approach**. This approach has been criticized for overestimating indirect costs, as labor can be replaced 36, 37 and there is continued debate regarding which approach is most appropriate. However, as the number of dengue-related deaths was relatively low [6] (Table S1), the estimated total cost associated with these fatal cases only accounted for a small part of the overall economic burden (<4%). Consequently, in this case a different approach would have little impact on the estimated total economic burden. In contrast, dengue-related deaths are highly significant in terms of the estimated DALY burden (Table S2).

The primary reason for the higher recent estimate from Shepard *et al*. [6] is that the estimates of the number of symptomatic dengue cases occurring in Vietnam have notably increased (Table 2 and Figure 1B). This was largely due to the updated methods used for adjusting for the underreporting of symptomatic dengue cases. In contrast, the estimate from Stahl *et al*. [30] was notably lower than the other estimates, most likely because it was based on the number of reported symptomatic dengue cases and did not adjust for underreporting.

**Table 2:** Summary of the Different Estimates of the Total Annual Cost of Dengue Illness in Vietnam (2016 US$ prices)

Study	Total cost of illness (US$ millions)	Assumed number of symptomatic dengue cases
Stahl *et al*. [30]	US$5.43^a^	69 680^b^
Shepard *et al*. [9]	US$29.77	442 911
Luong *et al*. [29]	US$46.55 (pertaining only to southern Vietnam)	413 411
Shepard *et al*. [6]	US$94.87	2 263 880

a We did not include the estimated costs related to vector control, surveillance, and IEC (information, education, and communication) within this value – as these are related to the costs of dengue control and not the cost of dengue illness itself.

b This estimate is based on the number of reported symptomatic dengue cases and did not adjust for underreporting. The results have been adjusted to 2016 prices.

## Data Gaps and Future Research Needs

In the following section, we summarise key future research needs for refining the economic burden estimates of dengue, focusing particularly on Vietnam. Many of these issues have also been highlighted in other papers on dengue 6, 30, 38, 39, 40, 41.

### Estimates of the Number of Symptomatic Cases

A key component of estimating the economic burden of dengue is approximating the number of symptomatic cases. There are several areas of further research that could improve the current estimates (outlined further in 40, 42, 43).

A vital area of future research for this is conducting comprehensive cohort studies in a range of different settings across Vietnam, with active dengue surveillance over a number of years. These will allow updated expansion factors to be calculated. When interpreting these studies, it will be necessary to account for the fact that the per-capita incidence of dengue may vary across different settings [44], which affects the generalizability of the studies. In addition, research has shown that reporting rates can vary with each dengue season, and that they can be affected by outbreaks of other diseases (such as malaria) 45, 46.

These cohort studies also need to collect more data regarding the type of care dengue cases seek, particularly regarding the different sources of outpatient care and the number of cases treated both within and outside the professional healthcare sector. These studies need to also investigate how variable this type of data is across the different study sites and account for the cases treated within the private healthcare sector. These data will allow the proportions of the different types of dengue cases considered in cost of illness studies to be estimated more accurately. Further investigation of the number of dengue-related deaths that are misclassified will also be important.

Despite their importance, conducting these types of studies is costly, time-consuming, and not always feasible in a large number of settings. Shepard *et al*. [40] highlighted that a promising strategy for future studies would be to adopt a ‘portfolio’ approach, where a combination of different approaches and data sources are combined. For example, the results from cohort studies in specific areas can be extrapolated using modeling accounting for the relevant covariates. This type of approach could be highly variable for improving the estimates of the number of symptomatic dengue cases in Vietnam. When using retrospective data, it is important to note that, in 2009, the WHO updated the dengue case classification [4]. This makes comparing data across studies before and after 2009 more problematic.

Having more accurate estimates of the number of symptomatic cases is not only vital for estimating the health and economic burden of dengue, but also for performing cost-effectiveness analyses of future dengue interventions.

### Cost of Illness Data

Though we identified several studies reporting the cost of dengue care in Vietnam, there are still data gaps that need to be filled to allow for more refined economic burden estimates. These are summarized in Box 3.

Box 3 **Key Data Gaps Regarding Dengue Cost of Illness Data**

**1. Data from a Wider Range of Settings**

The majority of the costing studies we identified were performed in Ho Chi Minh City. However, it is conceivable that the costs are different in more rural settings. Future analyses also need to collect data from hospitals at different levels of the health system (such as central hospitals, provincial hospitals, and district hospitals).

**2. More Cost Data Related to Outpatient Care**

Currently, there is inadequate information regarding the costs of dengue outpatient care in Vietnam. It will be important that future studies investigate the costs of different types/sources of outpatient care.

**3. Costs Related to Treatment Outside the Formal Healthcare Sector**

Currently there is very little information regarding the costs of patients self-treating.

**4. Updated Estimates since the Changes to Many of the Standard Healthcare Charges**

There have recently been changes to many of the standard health care charges within the Vietnamese health system. Therefore, the costs of dengue care have likely changed, and the estimates need to be updated.

**5. Stratifying the Cost Estimates by the Payer**

It would be beneficial if future studies stratified the costs by the payer. This is particularly important for the direct medical cost category, which can be split between the governmental health insurance system and the patient.

**6. Indirect Costs (Productivity Costs)**

We identified notable variation in how the indirect costs were estimated. The key sources of variation were:

• Whether the productivity losses of the patients’ informal caregivers were quantified.
• Whether or not lost unpaid work (such as household chores or childcare) was assigned an economic value. This can be particularly important for valuing the burden on informal caregivers [85].
• What wage source was used to value lost productivity. It should be noted that the WHO-CHOICE report stated that per capita GDP may overestimate the marginal product of labor [86].
• The time period investigated. Whether the indirect costs associated with healthcare that the patients received before they are hospitalized or after they were discharged were quantified.
• The specific methodology and questions used to ask patients about their indirect costs. Were patients asked directly how much income they lost due to the illness, or were they asked how much time they lost which was subsequently assigned an economic value?
• Whether missed school days were assigned an economic value.

In future studies, the methods used to estimate the indirect costs should be more clearly stated, allowing for more consistency and greater comparability.

Alt-text: Box 3

The comparability of the reviewed studies was restricted due to variations in the perspective taken, what cost items were included, as well as how the results were reported [39]. To improve the comparability of different studies, guidelines for estimating dengue-related costs have been proposed 39, 47. It would also be beneficial if future studies clearly report the exchange rates used, the year of the prices, and specifically state the approach used to adjust for inflation [48].

The current economic burden estimates do not account for the potential burden and income loss associated with symptoms that persist after resolution of the acute dengue episode, such as depression, profound fatigue, and weight loss 26, 27, 49, 50. A study in Mexico indicated that accounting for these persistent symptoms could increase the estimated economic burden of dengue by approximately 13% [27]. More data would be useful in supporting better estimation of this burden in Vietnam.

### Seasonality and Implications for Other Infections

Dengue outbreaks are typically seasonal, with the majority of cases occurring over a period of several months. This means that hospitals, particularly intensive care wards, may become congested during dengue outbreaks. It is possible that this could have negative consequences on care for patients with other conditions due to deterioration of overall service quality, an effect which has not yet been quantified. Accurately quantifying the extent of this burden would likely require intensive prospective studies.

It is also important to consider potential effects of dengue infection in individuals with comorbidities, such as diabetes, stroke, and renal disease [51]; not only is the dengue episode more likely to be severe, but also there may be destabilisation of the comorbid condition, both situations being likely to increase the costs of treatment [39]. Future studies are needed to investigate these interactions and how they may influence the cost of dengue illness over time.

### The Impact on Tourism

To our knowledge, there is currently limited information regarding lost revenue from tourism due to dengue 52, 53. A recent study estimated that, if dengue were eradicated, tourist expenditure would increase by US$2.86 billion in the affected countries – price year unclear [53]. More research is needed to estimate the potential negative impact of dengue outbreaks on tourism in Vietnam.

### Evaluating the Cost and Cost-Eeffectiveness of Dengue Interventions

There is very little published information regarding the cost of dengue control interventions in Vietnam. It will be important for future studies to evaluate the cost and cost-effectiveness of both the current and novel future interventions 54, 55, such as the Dengvaxia*^®^* vaccine 56, 57, 58.

One review reported that the government budget for vector control was US$5.57 million in 2011, with an additional US$1.08 million budgeted for surveillance and US$0.58 million budgeted for information, education, and communication (all in 2016 prices) [30]. Previous estimates of the cost of the vector control program were lower but still substantial 29, 59. However, the cost of dengue control measures will vary year by year, depending on the size and location of outbreaks. At times, it is also possible that the national budget for vector control will be supplemented at local administration levels, making it more complicated to estimate the overall cost of dengue control. Unless the expenditure from the different sources is collected, overall costs will likely be underestimated.

Beatty *et al*. [54] and Constenla *et al*. [38] found that there was very little consistency in the way costs of dengue interventions were evaluated, making generalizations and comparisons difficult. Moving forward it would be beneficial if studies were to adhere to standardized guidelines (such as CHEERS [60]) regarding what should be reported within the manuscript.

## Concluding Remarks

Dengue interventions are currently of great interest to policymakers around the globe, and in the coming years cost-effectiveness analyses will have a vital role in informing dengue policy decisions. Accurate estimations of the health and economic burden of dengue will be key components of these analyses.

The health and economic burdens of dengue in Vietnam have been consistently estimated to be substantial; the most recent studies have estimated that, in Vietnam, dengue is responsible for 39 884 DALYs annually and that the cost of dengue illness is approximately US$94.87 million per year in 2016 prices. However, we found that these types of estimates were highly sensitive to assumptions regarding the total number of symptomatic dengue cases that occur. Furthermore, we found that the DALY calculation for dengue has changed significantly over the last decade.

We identified several important data gaps and research needs that must be addressed to allow for more refined health and economic burden estimates in the future (see Outstanding Questions). Of particular importance are improved estimates regarding the number of symptomatic dengue cases/dengue-related deaths, more cost data related to the different types of outpatient care, and greater consistency regarding how indirect costs are quantified and reported.

Outstanding Questions Is it possible to estimate more accurately the true number of symptomatic dengue cases occurring?

What are the post-acute consequences of dengue, and how do they influence its health and economic burden?

How much does the economic burden of outpatient dengue cases vary depending on the different types/sources of outpatient care?

Can future studies use a more constant methodology for estimating the indirect (productivity) costs associated with dengue cases?

## Glossary

**Direct costs**: represent the costs of the goods, services, and other resources consumed in providing and accessing health care. These can be divided into two key types:
Direct medical costs: include the value of goods and resources directly related to medical services/care (such as the physician services, diagnostic tests, and drugs).
Direct nonmedical costs: include the value of resources related to the consumption of nonmedical resources (such as the patients’ transportation costs, and food expenses).
**Disability-adjusted life years (DALYs)**: DALYs are a measure of disease burden and are calculated as the sum of the years of life lost due to premature mortality and the years of healthy life lost due to disability. One DALY can be thought of as 1 year of ‘healthy’ life lost.
**Disability weight**: the number of years of healthy life lost due to disability are calculated using a disability weight factor (which is between 0 and 1) that reflects the severity of the disease.
**Expansion factor**: officially reported dengue case numbers are often adjusted for underreporting by using an expansion factor (the ratio of the estimated true number of symptomatic dengue cases to the reported number of dengue cases).
Fatal cases: patients who die due to dengue.
**Human capital approach**: the human capital approach takes the patient's perspective for valuing lost productivity and therefore counts any hour not worked by the patient as an hour lost. In this context it estimates the economic value of years of life lost due to premature mortality based on the discounted present value of that person’s expected economic productivity.
**Indirect costs (also known as productivity costs)**: represent the value of the productivity losses that result from illness, treatment, or premature death. These indirect costs can also include the productivity losses incurred by informal caregivers (who may have lost work when accompanying the patient to receive care, or when caring for them at home). It should be noted that there is significant variation in how indirect costs are estimated 36, 85, 87.
Symptomatic dengue cases: cases that are symptomatic. Within cost of illness studies, symptomatic dengue cases are often divided into one of the following mutually exclusive categories:
Nonfatal cases that are hospitalized: patients who are hospitalized due to their dengue infection.
Nonfatal cases that are treated in an outpatient (ambulatory) setting: patients who consulted a health professional for care, but who were not hospitalized (such as cases treated in outpatient departments, health centers/clinics, and clinicians’ private offices).
Nonfatal cases that are treated outside the formal healthcare sector: patients who did not receive diagnosis/treatment from a health facility but who may have purchased therapeutic products or diagnostic tests at their own initiative.

## Resources

^i^www.who.int/mediacentre/factsheets/fs117/en/

^ii^www.wpro.who.int/vietnam/topics/dengue/factsheet/en/

^iii^www.healthdata.org/gbd/data-visualizations
