# Supplementary material for: The Estimates of the Health and Economic Burden of Dengue in Vietnam
Source: Trends Parasitol. 2018 Oct;34(10):904–18. doi: 10.1016/j.pt.2018.07.007 (PMC6192036; doi:10.1016/j.pt.2018.07.007)
Supplement: Supplementary file 1 [file mmc1.pdf]

The estimates of the health and economic burden of dengue in Vietnam

Supplemental Information

The estimates of the health and economic burden of dengue in Vietnam

Trinh Manh Hung[1], Hannah E Clapham[1,2], Alison A Bettis[3,4], Hoàng Quốc Cường[5], Guy E Thwaites[1,2], Bridget A Wills[1,2], Maciej F Boni[6], Hugo C Turner[1,2]*

Correspondence: hturner@oucru.org

Overview of the adjustments for inflation

In order to directly compare the costs reported by the different studies (performed at different times), it was necessary to adjust for inflation. To do this, all the costs reported in US dollars were first converted back to Vietnamese dong (₫) – using either the exchange rate reported by the authors or the average exchange rate for the year the costs were collected. The costs were then adjusted for inflation using Vietnamese GDP implicit price deflators - which were extracted from the International Monetary Fund (IMF) [S1]. This was done by dividing the GDP deflator from 2016 by the GDP deflator for the year the cost data was collected. This ratio was then multiplied with the original costs in Vietnamese dong to adjust the costs to their 2016 value. Finally, these values were re-converted from Vietnamese dong to US dollars using the average Vietnam dong to dollar exchange rate from 2016 (US$1 = 21,935₫) [S2].

We used the Vietnamese GDP deflator rates to adjust the costs for inflation, as we believed they were a better measure for the price change that has occurred then the US inflation rates. This is consistent with the recommendations outlined within [S3].

A limitation of this approach is that the Vietnamese GDP deflator rates may not be truly reflective of price level changes for all of the resources used to provide care for a dengue case. In particular, the costs of the tradable goods (i.e. a good that can be sold in a different country from where it was produced) will likely not have changed in price to the same degree. Therefore, using this method could overestimate the inflated costs relating to these resources.

In addition, there were occasional differences between the exchange rate used by a study and the reported average for that year. However, generally the impact of this was negligible.

Other modifications and assumptions

Harving *et al.* [S4]:

As the dong to dollar exchange rate used within the study was not stated, we used the average exchange rate for 2005 [S2]. Within the study, the patients' transportation and food cost were categorized as an indirect cost. To make the results more comparable with other studies we re-categorized these costs as direct non-medical.

Luong *et al.* [S5]:

Because the original data from Luong *et al.* [S5] was not available, we based our costs on the summarized values reported by Shepard *et al.* [S6]. Based on the stated values from other costing studies within Shepard *et al*. [S6], we inferred that they had adjusted for inflation using United States (US) inflation rates. We therefore re-converted the summarized values (which were in 2010 prices) to their original values by:

1. Using the US GDP deflators to re-convert the values from 2010 to the original 2007 prices (i.e. undoing the previous adjustment for inflation) [S7]
2. Using the average dong-to-dollar exchange rate from 2007, which was reported in Luong *et al.* study [S5], to reconvert the values into Vietnamese dong.

We then adjusted for inflation using the method used for the other studies (i.e. using Vietnam-specific inflation rates from 2007 to 2016, and then converted to US dollars using average exchange rate from 2016).

Lee *et al*. [S8]:

Within the study, the original cost data for Vietnam was collected in 2012 and the reported values were adjusted to 2014 prices using Consumer Price Index deflators from Vietnam. The costs were then converted to US dollars using the 2014 exchange rate. We therefore converted the reported values back to Vietnamese dong (using the official exchange rate from 2014 [S2]) and then adjusted the values back to their original 2012 prices (using Consumer Price Index deflators for Vietnam) [S9]. The costs were then adjusted to 2016 US dollar prices using the same methodology outlined above.

Supplementary Box S1: Adjusting for underreporting and expansion factors.

Officially-reported dengue case numbers are often adjusted for underreporting by using an **expansion factor** (the ratio of the estimated true number of symptomatic dengue cases to the reported number of dengue cases).

Several studies have estimated local expansion factors in Vietnam ranging between 3.4 and 6.2 [S10, S11, S12, S13] i.e., in these specific areas within Vietnam the estimated true number of symptomatic dengue cases was between 3.4 and 6.2 times the reported number. It is noteworthy that, compared with estimates from other countries in Southeast Asia, these expansion factors were amongst the lowest identified [S12, S13, S14]. Furthermore, most of the studies were based in southern Vietnam and it is uncertain how generalizable these estimates are to the northern provinces of Vietnam.

In contrast, Toan *et al*. [S14] estimated national expansion factors for Vietnam which were substantially higher – ranging between 15 to 33. Their estimates for Vietnam were similar to those for Cambodia (11-22) and the Philippines (14-15) but lower than those for Thailand (11-82), and Indonesia (36-126) [S14]. Within their study, Toan *et al*. [S14] found that their estimated national expansion factors were consistently higher than their local expansion factors (with a few exceptions). They highlighted that this could be for the following reasons:

1. Performing an active surveillance study in a given area may unintentionally influence the number of cases that are reported through the routine surveillance system [S14].
2. Site-selection bias in cohort studies. Studies are often performed in areas where high dengue transmission is expected to occur. Therefore, the incidence of dengue within the study sites is likely to be higher than average [S14].
3. Differences in the methods for case detection and case definitions [S14].

Supplementary Table S1: Key estimates regarding the total number of symptomatic dengue cases and dengue-related deaths occurring annually in Vietnam (related to Figure 1B)

Study	Hospitalized cases per year	Cases treated in outpatient settings per year	Cases treated outside the formal healthcare sector per year	Fatal cases per year (95% CI)	Total number of symptomatic cases per year (95% CI/UI)
Shepard *et al.* [S6] (based on [S13])	81,611 (80,001-218,672)	361,300 (202,851-369,179)	-	80 (82-224)	442,911 (397,859-470,849)
Shepard *et al.* [S15] (based on [S16] [a])	387,576	1,715,836	160,390	78	2,263,880
Bhatt *et al.* [S17]	-	-	-	-	2,603,443[b] (1,890,174-3,578,852)

[a] *Note that though the Shepard et al. [S15] estimates were based on the GBD 2013 study [S16], there were some differences in the methods and the reported estimates.* [b] *Bhatt et al. [S17] also estimated that in 2010, there were 7,965,912 (95% CI: 6,081,413-10,371,255) asymptomatic dengue infections in Vietnam. The Shepard et al. [S6] estimates are representative of the period between 2001-2010. The Shepard et al. [S15] estimates are based on the GBD 2013 study [S16]. The Bhatt et al. [S17] estimate pertains to 2010. The different case categories are defined in the Glossary. CI: Credible Interval UI: Uncertainty Interval.*

Supplementary Table S2: The different estimates of the total number of disability-adjusted life years due to dengue in Vietnam annually.

Study	Years of healthy life lost due to disability (95% UI)	Years of life lost due to premature mortality (95% UI)	Disability-adjusted life years (95% CI/UI)
Shepard *et al.* [S6]	Not reported	Not reported	11,079 (7,226–16,452)
GBD 2013 [S16]	21,877 (7,515-54,111)	18,008 (9,374-27,202)	39,884 (22,571-73,760)

GBD: Global Burden of Disease. UI: Uncertainty Interval. CI: Credible Interval. The Shepard et al. [S6] estimates are representative of the period between 2001-2010. The model used within the GBD 2013 [S16] study smoothed out the effects of dengue outbreaks, making the estimate more representative of an average year around 2013.

Supplementary Table S3: The assumed number of years of life lost at different ages of death under the different GBD studies and assumptions

Age range	GBD 1990 (with age-weighting and discounting)		GBD 1990 (without age-weighting and discounting)		GBD 2010
	**Male**	**Female**	**Male**	**Female**	**Persons**
Neonatal	33.27	33.38	79.94	82.43	86.01
Post-neonatal	34.22	34.34	78.85	81.36	85.68
1-4	35.17	35.29	77.77	80.28	83.63
5-9	37.22	37.36	72.89	75.47	78.76
10-14	37.31	37.47	67.91	70.51	73.79
15-19	36.02	36.22	62.93	65.55	68.83
20-24	33.84	34.08	57.95	60.63	63.88
25-29	31.11	31.39	52.99	55.72	58.94
30-34	28.08	28.40	48.04	50.83	54.00
35-39	24.91	25.30	43.10	45.96	49.09
40-44	21.74	22.19	38.20	41.13	44.23
45-49	18.63	19.16	33.38	36.36	39.43
50-54	15.65	16.26	28.66	31.68	34.72
55-59	12.82	13.52	24.07	27.10	30.10
60-64	10.19	10.96	19.65	22.64	25.55
65-69	7.80	8.60	15.54	18.32	21.12
70-74	5.71	6.45	11.87	14.24	16.78
75-79	4.00	4.59	8.81	10.59	12.85
80-84	2.68	3.09	6.34	7.56	9.34
85+	1.37	1.23	3.82	3.59	5.05

Adapted from [S18]. GBD: Global Burden of Disease.

Supplementary Table S4: Summary of the cost of dengue illness in Vietnam estimate from the Shepard *et al*. [S15] study (2016 US$ prices).

	Non-fatal cases			Fatal cases	Total
	**Hospitalized**	**Treated in outpatient settings**	**Treated outside the formal healthcare sector**		
**Assumed unit cost per case (US$)**					
Direct cost	67.74	23.25	5.05	NA	-
Indirect cost	13.14	10.11	10.11	Child: 55,604<br>Adult: 36,395	-
**Total**	**80.88**	**33.36**	**15.16**	**-**	-
**Estimated annual economic burden (US$)**					
Direct cost	26,253,412	39,456,193	776,545	NA	**66,484,150**
Indirect cost	5,258,518	17,983,517	1,681,036	3,466,490	**28,389,149**
**Total**	**31,511,518**	**57,437,709**	**2,457,582**	**3,466,490**	**94,873,299**

The data were adapted from Shepard et al. [S15]. The estimated number of cases (stratified by case type) are shown in Figure 1B and Supplementary Table S1. Within this study [S15], symptomatic dengue cases were divided into four mutually-exclusive categories, depending on care sought, which are defined in the Glossary. The results have been adjusted to 2016 prices. NA: Not Applicable.

Supplementary Table S5: Reported costs related to hospitalized and outpatient dengue cases in international dollars (2016 prices)

Study	The sample	Direct medical costs per case (Int$)	Direct non-medical costs per case (Int$)	Indirect costs per case (Int$)	Total cost per case (Int$)
**Hospitalized cases**					
Harving *et al.* [S4]	The families of children hospitalized with dengue hemorrhagic fever at a hospital in Ho Chi Minh City were interviewed regarding their out-of-pocket expenses.	178.08 *(excluding the costs covered by insurance schemes)*	86.24	69.53	**333.85**
Tam *et al.* [S19]	Dengue hemorrhagic fever patients admitted to two hospitals in Can Tho province were interviewed 6–9 months after their recovery.	371.25	249.61	185.63	**806.49**
Luong *et al.* [S5]	A multicenter cost study in four hospitals in southern Vietnam. Both urban and rural settings were sampled.	208.29	75.90	56.91	**341.10**
Pham *et al.* [S20]	Patients from one hospital in a suburban area of Ho Chi Minh City were interviewed. Direct medical costs were also collected from the hospital's electronic database.	135.97	118.65	147.23	**401.85**
Stahl *et al.* [S21]	A sample of patient records collected from one hospital.	131.45 *(excluding staff costs)* [a]	NA	NA	**NA**
Vo *et al.* [S22]	Direct medical costs were collected from the electronic database from a hospital in Ho Chi Minh City.	142.51	NA	NA	**NA**
Lee *et al.* [S8]	Hospitalized patients from the Khanh Hoa General Hospital (Khanh Hoa province).	225.05	138.29	181.67	**545.01**
**Outpatient (ambulatory) cases**					
Luong *et al.* [S5]	A multicenter cost study in four hospitals in southern Vietnam. Both urban and rural settings were sampled.	43.75	52.72	43.97	**140.43**
Stahl *et al.* [S21]	A sample of patient records collected from one hospital.	82.54	NA	NA	**NA**
Lee *et al.* [S8]	Suspected dengue cases visiting the outpatient clinic at Khanh Hoa General Hospital (Khanh Hoa province).	70.48	24.40	73.21	**168.09**

[a] *Also reported the following costs (2016 prices): Int$162.02 per adult general ward inpatient; Int$140.63 per child general ward inpatient; Int$330.16 per intensive care unit patient.*
The cost categories are defined in the Glossary.
All of the presented results have been adjusted to international dollars (2016 prices). The purchasing power parity conversion factor for 2016 was retrieved from the World Bank (Int$1 = 7,562.939 VND) [S23].
NA: Not Available, Int$: International Dollar.

Supplementary references

S1 International Monetary Fund (2017) Vietnam: Gross domestic product, deflator (Index).
S2 World Bank (2017) Official exchange rate.
http://data.worldbank.org/indicator/PA.NUS.FCRF?locations=VN&page=6
S3 Kumaranayake, L. (2000) The real and the nominal? Making inflationary adjustments to cost and other economic data. *Health Policy Plan* 15, 230-234
S4 Harving, M.L. and Rönsholt, F.F. (2007) The economic impact of dengue hemorrhagic fever on family level in Southern Vietnam. *Dan Med Bull* 54, 170-172
S5 Luong, Q.C.*, et al.* (2012) Assessing the economic burden of dengue in Southern Viet Nam: Results of a prospective multicenter cost study. Pasteur Institute
S6 Shepard, D.S.*, et al.* (2013) Economic and Disease Burden of Dengue in Southeast Asia. *PLoS Negl Trop Dis* 7, e2055
S7 International Monetary Fund (2016) United States: Gross domestic product, deflator.
http://www.imf.org/external/pubs/ft/weo/2016/02/weodata/weorept.aspx?sy=1990&ey=2021&scsm=1&ssd=1&sort=country&ds=.&br=1&pr1.x=77&pr1.y=13&c=111&s=NGDP_D&grp=0&a=
S8 Lee, J.S.*, et al.* (2017) A multi-country study of the economic burden of dengue fever: Vietnam, Thailand, and Colombia. *PLoS Negl Trop Dis* 11, e0006037
S9 World Bank (2017) Consumer Price Index, Vietnam.
https://data.worldbank.org/indicator/FP.CPI.TOTL?locations=VN
S10 Tien, N.T.K.*, et al.* (2010) A prospective cohort study of dengue infection in schoolchildren in Long Xuyen, Viet Nam. *Trans R Soc Trop Med Hyg* 104, 592-600
S11 Phuong, H.L.*, et al.* (2006) Dengue as a cause of acute undifferentiated fever in Vietnam. *BMC Infect Dis* 6, 123
S12 Nealon, J.*, et al.* (2016) Symptomatic Dengue Disease in Five Southeast Asian Countries: Epidemiological Evidence from a Dengue Vaccine Trial. *PLoS Negl Trop Dis* 10, e0004918
S13 Undurraga, E.A.*, et al.* (2013) Use of expansion factors to estimate the burden of dengue in Southeast Asia: a systematic analysis. *PLoS Negl Trop Dis* 7, e2056
S14 Toan, N.T.*, et al.* (2015) Dengue epidemiology in selected endemic countries: factors influencing expansion factors as estimates of underreporting. *Trop Med Int Health* 20, 840-863
S15 Shepard, D.S.*, et al.* (2016) The global economic burden of dengue: A systematic analysis. *Lancet Infect Dis* 16, 935-941
S16 Stanaway, J.D.*, et al.* (2016) The global burden of dengue: an analysis from the Global Burden of Disease Study 2013. *Lancet Infect Dis* 16, 712-723
S17 Bhatt, S.*, et al.* (2013) The global distribution and burden of dengue. *Nature* 496, 504-507
S18 World Health Organization (2013) WHO methods and data sources for global burden of disease estimates 2000-2011
S19 Tam, P.T.*, et al.* (2012) High household economic burden caused by hospitalization of patients with severe dengue fever cases in Can Tho province, Vietnam. *Am J Trop Med Hyg* 87, 554-558
S20 Pham, L.D.*, et al.* (2016) Economic report on the cost of dengue fever in Vietnam: case of a provincial hospital. *Clinicoecon Outcomes Res* 9, 1-8
S21 Stahl, H.C.*, et al.* (2013) Cost of dengue outbreaks: literature review and country case studies. *BMC Public Health* 13, 1048
S22 Vo, N.T.T.*, et al.* (2017) Direct medical costs of dengue fever in Vietnam: A retrospective study in tertiary hospital. *Malaysian Journal of Medical Sciences* 24, 66-72
S23 World Bank (2018) PPP conversion factor, GDP (LCU per international $).
https://data.worldbank.org/indicator/PA.NUS.PPP?locations=VN
