# Supplementary material for: The Estimates of the Health and Economic Burden of Dengue in Vietnam
Source: Trends Parasitol. 2018 Oct;34(10):904–18. doi: 10.1016/j.pt.2018.07.007 (PMC6192036; doi:10.1016/j.pt.2018.07.007)
Supplement: Supplementary file 2 [file mmc2.docx]

**Supplemental Information Inventory**

Box S1 shows the variation in the expansion factors that have been estimated for Vietnam.

Table S1 summarizes the data presented in Figure 1B regarding the estimates of the true number of symptomatic dengue cases.

Table S2 summarizes the different estimates regarding the total number of disability-adjusted life years due to dengue in Vietnam annually.

Table S3 summarizes the assumed number of years of life lost at different ages of death under the different GBD studies.

Table S4 provides a detailed summary of the estimated total cost of dengue illness in Vietnam from of Shepard *et al*. (2016) study*.*

Table S5 reports the costs related to hospitalized and outpatient dengue cases in international dollars.
